# Cytotoxicity and Identification of Antibacterial Compounds from *Baillonella toxisperma* Bark Using a LC-MS/MS and Molecular Networking Approach

**DOI:** 10.3390/metabo13050599

**Published:** 2023-04-27

**Authors:** Morel Essono Mintsa, Brice Serge Kumulungui, Cédric Sima Obiang, Elodie Dussert, Elodie Choque, Damien Herfurth, Rozenn Ravallec, Joseph-Privat Ondo, François Mesnard

**Affiliations:** 1 UMRt BioEcoAgro 1158-INRAE, BIOPI, Université de Picardie Jules Verne, 1 Rue des Louvels, 80000 Amiens, France; 2 Centre Interdisciplinaire de Recherches Médicales de Franceville (CIRMF), Franceville P.O. Box 769, Gabon; 3 Laboratoire Innovation Matériau Bois Habitat (LIMBHA), Ecole Supérieure du Bois, 7 Rue Christian Pauc, 44306 Nantes, France; 4 Laboratoire de Recherches en Biochimie (LAREBIO), Université des Sciences et Techniques de Masuku, Franceville P.O. Box 943, Gabon; 5 UMRt BioEcoAgro 1158-INRAE, Institut Charles Violette, Université de Lille, 59655 Lille, France

**Keywords:** antibacterial activity, *Baillonella toxisperma*, cytotoxicity, dereplication, molecular networks

## Abstract

*Baillonella toxisperma* is a medicinal plant used in northern Gabon to treat microbial diseases. It is a plant well-known by local populations, but very few studies have focused on the molecules responsible for the antibacterial activities of *B. toxisperma*. This study proposes a dereplication strategy based on molecular networking generated from HPLC-ESI-Q/TOF data, allowing investigation of the molecules responsible for the antibacterial activity of *B. toxisperma*. From this strategy, eighteen compounds were putatively identified. All of these compounds belonged mainly to five families of natural compounds, including phenylpropanolamines, stilbenes, flavonoids, lignans and phenolic glycosides. The chemical study carried out from the bark of *B. toxisperma* allowed us to identify, for the first time, compounds such as resveratrol and derivatives, epicatechin, epigallocatechin and epigallocatechin gallate. In addition, antibacterial activity (diffusion method and microdilution) and cytotoxicity (Cell Counting Kit-8 (CCK-8 Assay)) in vitro were evaluated. The crude ethanolic extract, as well as the fractions of *B. toxisperma*, showed significant antibacterial activity. However, the ethanolic fractions F2 and F4 presented high antibacterial activity compared to the crude extract. Cytotoxicity studies on colon-cancer cells (Caco-2) and human keratinocyte cells (HaCaT) showed moderate cytotoxicity in both cell types. This study clearly shows the therapeutic potential of the ethanolic extract of the bark of *B. toxisperma* and provides information on the phytochemical composition and bioactive compounds of the plant.

## 1. Introduction

*Baillonella toxisperma* Pierre (*B. toxisperma*), commonly known as moabi in Cameroon and adzap in Gabon and Equatorial Guinea, is found wild in the forests of intertropical Africa [1]. Naturally durable, *B. toxisperma* is known for its pinkish-brown wood and the oil extracted from its fruit [2], which gives it multiple uses in traditional medicine. In the Cameroonian pharmacopoeia, decoctions of aqueous extracts of its bark are used in treatment of hemorrhoids, sexually transmitted infections, diarrhea, malaria, vaginal mycosis [3] and gastrointestinal and bronchopulmonary disorders [4]. In Gabon, this plant is used in traditional medicine in treatment of several pathologies, notably rheumatic pains, mycosis, wounds, ringworms and malaria. It is also used as an additive in other remedies [5]. *B. toxisperma* belongs to the Sapotaceae family, one of the most important plant families commonly distributed in the tropical forests of Africa, Australia and South America [6]. Indeed, Sapotaceae is one of the eight species of the richest family of angiosperms, it has about 1250 species and 58 genera [7,8]. Generally, the plants belonging to this family are known for their numerous pharmacological properties, as is the case for *B. toxisperma*. Since the 2000s, this plant has been the subject of several studies. The studies of Saha et *al*. showed strong antioxidant activity of the wood of the plant [4]. Other studies have also shown that the plant has antimicrobial and antifungal activities [9,10]. *B. toxisperma* also improves sexual performance and reproductive dysfunction induced by stress [3]. Although several biological studies have been conducted on the bark, fruit and leaves of *B. toxisperma* [11,12], very little work has been published on the compounds responsible for the biological activities of the plant. One of the few studies that isolated a compound (3-hydroxy uridine, a plant growth inhibitor) was performed by Hajime et al. [13]. Thus, in the present study, we propose a molecular networking (MN) dereplication approach based on high-resolution liquid chromatography/mass spectrometry (LC-HRMS/MS) data to more efficiently and rapidly target compounds present in the ethanolic extracts of *B. toxisperma* [14]. Molecular networking (MN) is a valuable tool for revealing the metabolomes of plants, humans, microorganisms and animals [15]. It can not only annotate compounds in complex matrices based on their tandem mass spectrometry (MS/MS) characteristics but also group them into molecular families based on their spectral similarities, thus facilitating visualization of structurally identical molecules within the same chemical family and structurally different molecules distributed in different chemical families [16,17]. This approach allows researchers to identify known compounds in order to focus on unknown compounds that may potentially be of biological interest [18]. The main purpose of our study was not only to have good visibility of the chemical composition of the bark of *B. toxisperma* but also to identify the molecules responsible for its antibacterial activity. Thus, a combined MN and LC-MS/MS approach was used. Considering the use of this plant in Gabonese traditional medicine, it was essential to test its innocuousness toward certain cell lines, including CaCo-2 cells and HaCaT cells. The structures of the compounds annotated in the ethanolic extract of *B. toxisperma* are mainly composed of phenolic compounds known for their antibacterial, antioxidant and antiproliferative activities on several cancer-cell lines [19,20,21,22]. To the best of our knowledge, the chemical composition and cytotoxic activities of *B. toxisperma* bark are described for the first time in this study.

## 2. Materials and Methods

### 2.1. Plant Material

Fresh trunk bark of *B. toxisperma* was collected in January 2019 from the village of Nvane Essabeigne in the Woleu-Ntem province of northern Gabon (0°43′0″ N, 11°37′60″ E). The botanical identification was confirmed by Professor Henri Paul Bourobou. A specimen (M.E.M 002) was deposited at the National Herbarium of Gabon (Libreville, Gabon).

### 2.2. Extraction and Fractionation

*B. toxisperma* bark was dried and then ground. From this powder (400 g), extraction by maceration under agitation was carried out for 24 h with 1500 mL of 70% ethanol. The extract obtained was filtered under a vacuum and then concentrated with a rotary evaporator to obtain 12.5 g of ethanolic crude extract. The crude extract was fractionated with Flash chromatography on an 80 g Reveleris® Grace Silica PF-50SIHC-F0080 cartridge (Rheinland-Pfalz, Germany) using a CH_2_Cl_2_ gradient, increasing the proportions of MeOH (100:0 to 0:100) with a fixed flow rate 60 mL/min [17]. The fractions obtained in the Flash chromatography were pooled with TLC thin-layer chromatography and were then analyzed in HPLC-QTOF-HRES to search for potential new compounds.

### 2.3. Data-Dependent LC-HR-ESI-MS^2^ Analysis

Data-dependent LC-HR-ESI-MS^2^ analysis was performed using a previously described method [17]. The extracts were analyzed using high-performance liquid chromatography coupled to a Sunfire® C18 analytical column (150 × 2.1 mm, 3.5 µm particle diameters; Waters, Milford, MA, USA). A standard elution gradient was used, with mobile phases A:H_2_O and B:MeOH, each containing 0.1% formic acid (HCOOH). Both solvents were eluted at a flow rate of 0.25 mL/min using a standard gradient of MeOH:H_2_O + 0.1% HCOOH (5:95 to 100:0 for 40 min). The temperature of the column oven was set to 40 °C and the injection volume to 5 µL. Detection was performed with a diode-array UV detector connected in series with an Agilent 6530 hybrid quadrupole time-of-flight mass spectrometer (Agilent Technologies, Massy, France) equipped with an ESI source, which was compatible with analytes with various polarity ranges. The ionization parameters were a 320 °C capillary temperature, a source voltage of 3500, a 10 L/min sheath-gas flow rate, 180 V fragmentation potential and 35 psi nebulizer pressure. The bypass valve was set to waste for the first 3 min. The analyzer provided satisfactory accuracy in the measurements, with a mass resolution of 11,000 at *m*/*z* 922, and the high-resolution measurements provided significant certainty in the calculated raw formulas for the observed fragment ions, this quality of data is necessary for the molecular network approach employed. MS scans were measured in the full-scan mode of *m*/*z*, between 100 and 1700, with a scan time of 0.1 s. The MS^1^ scan was followed by an MS^2^ scan of the three most intense ions above an absolute threshold of 5000 counts [17]. The selected parent ions were fragmented with a collision energy of 45 eV and an isolation window of 1.3 u.m.a [23]. The calibration solution contained two internal reference masses (Purine, C_5_H_4_N_4_, *m*/*z* 121.050873 and HP-921 [hexakis-(1H,1H,3H-tetrafluoropentoxy) phosphazene], C_18_H_18_O_6_N_3_P_3_F_24_, *m*/*z* 922.0098). A permanent MS/MS exclusion list criterion was established to avoid oversampling of the internal calibrant. LC-UV and MS data acquisition and processing were performed using MassHunter Workstation software (Agilent Technologies, Massy, France) [17]. The raw LC-HR-ESI-MS/MS data in .d format (Agilent) were converted to mzXML format with MSConvert software [24]. With MZmine 2 V32 software [25], all mzXML formats were processed. Then, the .mgf spectral data file and its .csv metadata file were exported to the GNPS web platform. A molecular network was created using the spectral clustering algorithm, with a cosine score of 0.65 and a minimum of six matched peaks. The molecular network data were analyzed and visualized using Cytoscape software (ver. 3.9.0) [26].

### 2.4. Bacterial Germs Tested

The bacterial carrier used in our study consisted of two reference bacterial strains (*Escherichia coli* ATCC 25922, *Escherichia coli* ATCC 8739) and three clinical strains, including multi-drug-resistant *Klebsiella pneumoniae*, extended-spectrum beta-lactamase-producing *Escherichia coli* and *Salmonella enterica*. All of these bacterial strains were obtained at the Centre Interdisciplinaire de Recherches Médicales de Franceville (CIRMF) in Gabon. 

### 2.5. Antibacterial Assays

The diffusion method was used to study sensitivity of microorganisms to plant extracts. Bacterial colonies were used to prepare the inoculum in order to have a density equivalent to that of 0.5 McFarland. Flood inoculation of the microbial suspension was performed, the agar was allowed to dry for 10 min. After drying, sterilized Wattman 1 paper disks impregnated with 20 µL of crude extract or fraction prepared at a concentration of 100 μg/mL (diluted in 1% DMSO) were placed on the Petri plates. Then, the latter were incubated for 18–24 h at 37 °C. Ticarcillin, tetracycline and gentamicin were used as positive controls.

The minimum inhibitory concentrations (MICs) of the crude extracts and fractions were determined with the microdilution method on 96-well microplates [27]. A series of seven dilutions of each extract (double dilutions ranging from 0.0049 to 5 mg/mL) were made in Muller–Hinton Nutrient Broth (Liofilchem, Roseto, Italy). To determine the minimum bactericidal concentrations (MBCs), nutrient agar was inoculated with 100 μL of the contents of the wells (concentrations greater than or equal to the MIC). The MBC was determined after a 24 h incubation at 37 °C. Antibacterial samples were considered to be bactericidal with MBC/MIC ratios equivalent to 1 or 2 and bacteriostatic if the MBC/MIC ratio was equivalent to 4 or 16 [27].

### 2.6. Cell Culture

Human colon epithelial cells, Caco-2 (accession N^o^ 86010202, European collection of authenticated cell cultures (ECACC), Salisbury, UK), were grown in monolayers in Dulbecco’s Modified Eagle’s Medium (DMEM), supplemented with 10% inactivated calcium-free fetal calf serum (Gibco Laboratories, Grand Island, NY, USA), 100 U/mL penicillin/0.1 mg/mL streptomycin (PanBiotech GmbH, Aidenbach, Germany) and 2 mM L- glutamine (PanBiotech GmbH, Germany), at 37 °C and in an atmosphere containing 5% CO_2_ (CO_2_ Incubator; Thermo Scientific, Marietta, OH, USA) [28]. HaCaT (immortalized human keratinocyte) cells (accession No. 300493, Cell Lines Service GmbH, Eppelheim, Germany) were cultured in DMEM medium that was low in calcium (0.03 mM) and supplemented with 10% calcium-free fetal calf serum, 100 U/mL penicillin/0.1 mg/mL streptomycin and 2 mM L-glutamine (PanBiotech GmbH, Germany). 

### 2.7. Cell Viability CCK-8 Assays

Cell viability was determined with the Cell Counting Kit-8 (CCK-8) assay (Dojindo Laboratories, Kumamoto, Japan) according to the manufacturer’s protocol [28]. Cells were seeded in 96-well plates at densities of 2 × 10^4^ cells/cm^2^ and 1.2 × 10^4^ cells/cm^2^ for the Caco-2 and HaCaT cells, respectively. After 4 days of culture for the HaCaT cells and 6 days of culture for the Caco-2 cells, the samples were diluted in DMEM (at increasing concentrations ranging from 100 to 500 µg/mL) and contacted with cells for 24 h at 37 °C (CO_2_ Incubator, Thermo Scientific, Marietta, USA) at a rate of 100 µL/well. WST-8 was then added to the final concentration of 5% for 2 h at 37 °C in the dark. The concentration of reduced WST-8 was measured at a 450 nm wavelength using the spectrophotometer (SpectraMax® iD3, Molecular Devices, San Jose, CA, USA) against “blanks or counter assays” containing all reagents for each condition except cells. Results are presented as percent viability compared to the control (DMEM 2% DMSO). Cell viability was calculated relative to the percentage of live cells in the DMSO control on GraphPad Prism 9.3.0 (463) using the following formula: % Cell viability = Mean OD of treated cells/Mean OD of DMSO control cells. The data were expressed as the mean ± standard deviation (SD) of triplicate independent experiments (N = 3 three independent experiments) for each line, in triplicate for each experiment (n = 9), and analyzed using a one-way analysis of variance (ANOVA), the Kruskal-Wallis test and Dunn’s post hoc test. * *p* = 0.0304; *** *p* = 0.0007 and **** *p* < 0.0001 were considered to be statistically significant.

## 3. Results and Discussion

### 3.1. Metabolite Profiling of B. toxisperma Bark from LC-MS/MS Analysis

**Figure 1:**
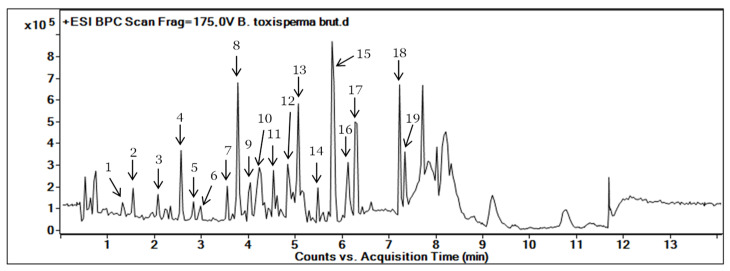
Base Peak Chromatogram (BPC) of the ethanolic extract of *B. toxisperma* obtained with HPLC-UV-MS/MS. The number on each peak corresponds to the numbers in Table 1.

Figure 1 shows the BPC (Base Peak Chromatogram) of the ethanolic extract of *B. toxisperma* obtained with HPLC-UV-MS in positive ion mode. This chromatogram allowed us to visualize the majority of the chromatographic peaks on broad polarity ranging from 5 to 100% of organic solvent.

The step of identification of the metabolites present in the ethanolic crude extract of the *B. toxisperma* trunk bark was carried out using a double identification (LC-MS/MS and molecular networks). These two identification steps allowed us not only to determine the elemental composition of the molecules present in the plant but also to have an overview of the families of molecules grouped in the plant. These two steps were made possible by exploiting high-resolution mass spectrometry data. The datasets were then annotated with the theoretical precise masses of the compounds contained in the public databases already described above. As in Table 1, from the crude extract of *B. toxisperma*, a total of nineteen compounds were putatively annotated. These metabolites mainly belong to five families of natural compounds: phenylpropanolamines, flavonoids, stilbenes, lignans and phenolic glycosides. Some of the major peaks were tentatively identified manually based on exact masses through comparison on public databases such as the Dictionary of Natural Products: https://dnp.chemnetbase.com (accessed on 22 October 2022) [29], PubChem and ChemSpider. Some of these compounds have also been identified in several other plants belonging to the family Sapotaceae [30]. Thus, on this basis, only compounds 1, 3, 4, 7, 8, 9, 10 and 11 were identified, as epigallocatechin; kelampayoside A; epigallocatechin gallate; resveratrol; β-conidendrin; 3,5-*O*-dimethyl resveratrol; methyl resveratrol and isorhapontin, respectively. 

The confidence levels of the compound annotations were performed with respect to the guidelines published by the Metabolomics Society [31]. Level 0, Unambiguous 3D Structure: the isolated, pure compound, including full stereochemistry; Level 1, Confident 2D Structure: uses reference standard-matching or full 2D structure elucidation; Level 2, Probable Structure: matched to literature data or databases with diagnostic evidence; Level 3, Possible Structure or Class: the most likely structure, isomers possible, substance class or substructure match; Level 4, Unknown Feature of Interest.

### 3.2. MS/MS–Molecular Networking-Based Dereplication

The analysis of the ethanolic crude extract of *B. toxisperma* was performed using the MN approach combined with LC-MS/MS as proposed on the GNPS website: http://gnps.ucsd.edu (accessed on 19 April 2022). All obtained HRESIMS/MS spectra were processed using MZmine2 software, thus, two types of files were obtained, in .mgf and _quant.csv formats. From these two files, the MS/MS spectra were searched in the spectral libraries hosted by the GNPS platform and the results were annotated as part of this dereplication strategy. To refine the structural information of the molecular network, an in silico structure annotation from GNPS Library Search was incorporated into the network using the MolNetEnhancer workflow [32].

**Figure 2:**
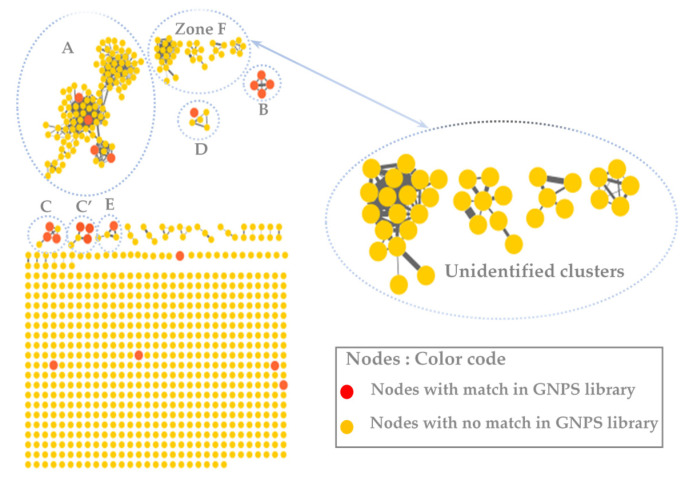
The molecular network of the ethanolic crude extract of *B. toxisperma* obtained with the GNPS platform and visualized with Cytoscape 3.9.0 software. The letters A, B, C, C,’ D and E represent different clusters with matches on the GNPS databases.

**Figure 3:**
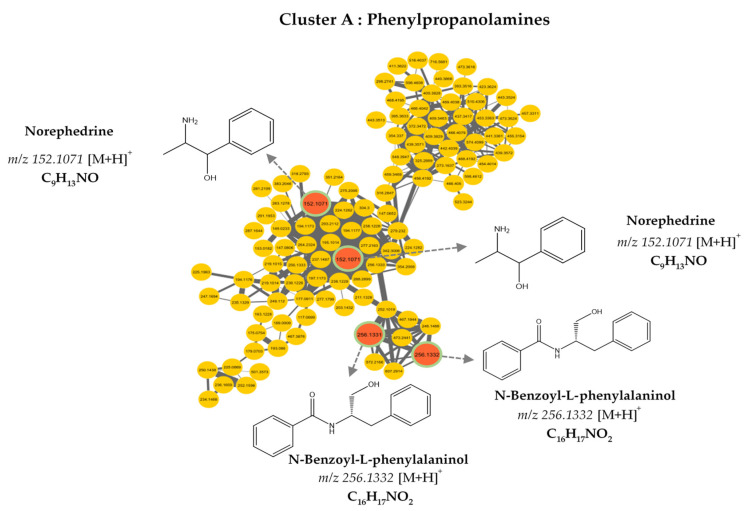

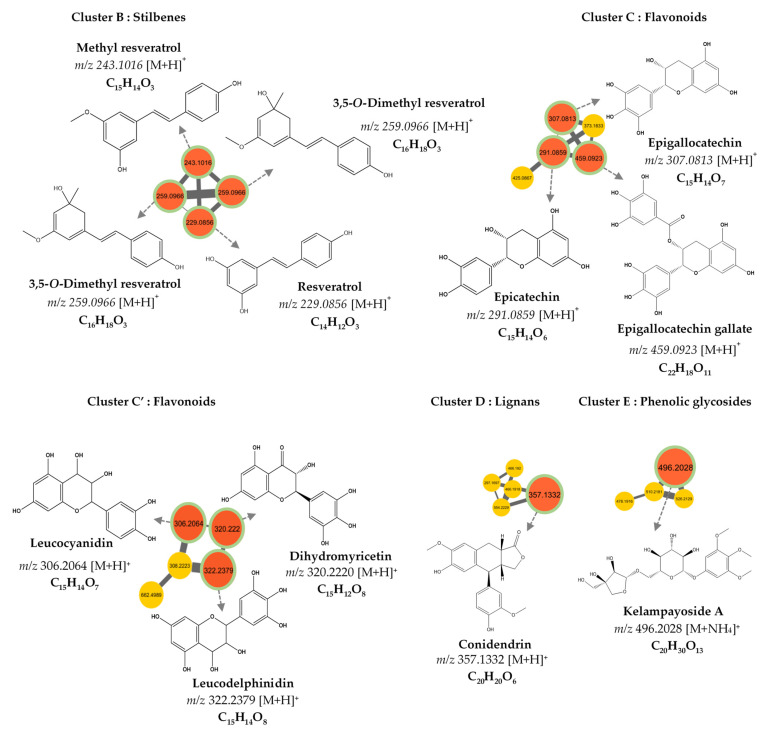
Molecular network analysis of the ethanolic crude extract of *B. toxisperma*. Nodes of clusters A (phenylpropanolamines), B (stilbenes), C, and C’ (flavonoids), D (lignans) and E (phenolic glycosides) were putatively annotated from the GNPS spectral library. Nodes in red represent nodes with a match in the GNPS library and those in yellow represent nodes with no match.

The molecular network was visualized using Cytoscape 3.9.0 software. This molecular network contained one large cluster and sixteen small clusters (number of nodes n ≥ 3) that could be linked into molecular families or groups (Figure 2)**.** As well as the manual exploitation of the MS/MS data, these results highlighted the presence of five major molecular families in the extract of *B. toxisperma*: phenylpropanolamines (cluster A), stilbenes (cluster B), flavonoids (cluster C and C’), lignans (cluster D) and phenolic glycosides (cluster E). Cluster B was composed of seven nodes, three of which had *m*/*z* 259.097 (C_15_H_14_O_3_); *m*/*z* 229.086 (C_14_H_12_O_3_) and *m*/*z* 243.102 (C_16_H_8_O_3_), corresponding to three stilbenes, respectively: 3,5-*O*-dimethyl resveratrol, resveratrol and methyl resveratrol. On the other hand, clusters C and C’ revealed six compounds of the flavonoid family, they are epicatechin (*m*/*z* 291.086; C_15_H_14_O_6_); epigallocatechin (*m*/*z* 307.081; C_15_H_14_O_7_); epigallocatechin gallate (*m*/*z* 459.092; C_22_H_18_O_11_); leucocyanidin (*m*/*z* 306.2064; C_15_H_14_O_7_); ampelopsin, still known as dihydromyricetin (*m*/*z* 320.2220; C_15_H_12_O_8_); and leucodelphinidin (*m*/*z* 322.2379; C_15_H_14_O_8_). Cluster-C compounds have been described by Baky et al. (2016) to be in several plants of the Sapotaceae family [30], and those of cluster C’ described by Ma et al. (2011) in a Fabaceae used in traditional Chinese medicine [33], which generally confirms the presence of these compounds at the level of angiosperms and, in particular, at the level of *B. toxisperma* (Figure 2 and Figure 3). Finally, phenylpropanolamines (cluster A) such as norephedrine (*m*/*z* 152.107; C_9_H_13_NO); N-benzoyl-L-phenylalaninol (*m*/*z* 256.133; C_16_H_17_NO_2_); lignans (cluster D), such as the example of conidendrin (*m*/*z* 357.369; C_20_H_20_O_6_) and phenolic glycosides (cluster E), such as kelampayoside A (*m*/*z* 496.203; C_20_H_30_O_13_), were also identified. Consistently with these results, the phenylpropanolamines appeared to be the largest family, with many associated nodes. Flavonoids, stilbenes and lignans were also well-represented in this ethanolic extract, with a fairly large cluster (number of nodes n ≥ 5), whereas phenolic glycosides were less numerous. Several compounds in cluster A were fragmented in the source, the fragment ions were then fragmented in MS/MS, which explains the redundancy of the information generated for this cluster with several nodes. Indeed, since the fragment ions generated in the source had MS/MS spectra very similar to the MS/MS spectrum of the parent ion, they were linked with a high degree of similarity (Table 2). As for the other clusters, they were not fragmented in the source, therefore, they contained nodes that represented only the molecular ions without information redundancy. We also noted the presence of four clusters where n ≥ 5, named zone F in Figure 2. In fact, the majority of the ion fragments present within these clusters could not be identified. This absence suggests that they are potentially new compounds.

### 3.3. Antibacterial Activity

**Figure 4:**
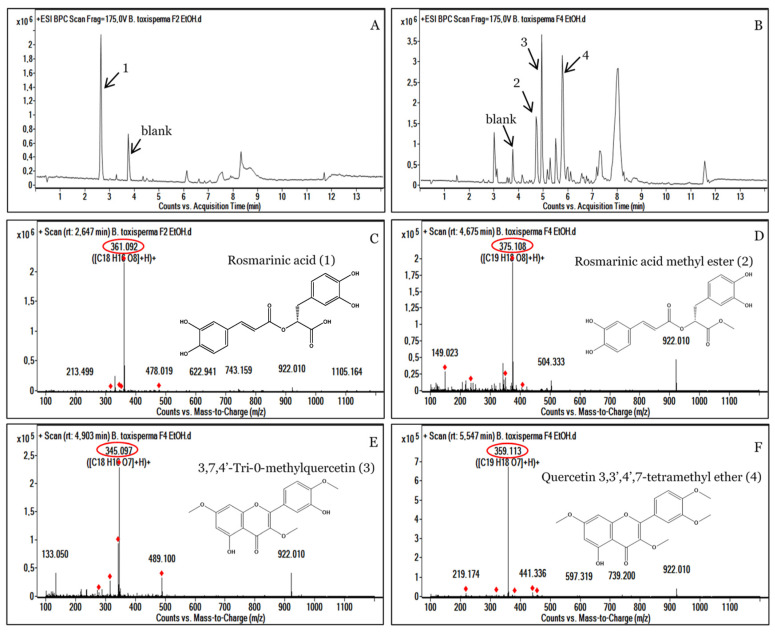
HPLC-UV-MS analyses of the active ethanolic fractions F2 (**A**) and F4 (**B**) of *Baillonella toxisperma* bark. Mass spectrum of [M+H]^+^ ions of rosmarinic acid (**C**); rosmarinic acid methyl ester (**D**); 3,7,4’,-trimethylquercetin (**E**) and quercetin 3,3’,4’,7-tetramethyl ether (**F**). The red circle represents the exact masses of the identified compounds.

The antibacterial activity of the ethanolic extract of *Baillonella toxisperma* was evaluated with five bacterial strains (*Escherichia coli* ATCC 25992, *Escherichia coli* ATCC 8739, multi-drug-resistant *Klebsiella pneumoniae*, *Salmonella enterica* and extended-spectrum beta-lactamase-producing *Escherichia coli*) with Gram negativity. The sensitivity tests of the plant extracts toward these microorganisms allowed us to determine the inhibition zone diameters (IZD) of the crude extract and the fractions. The results of the zones of inhibition are recorded in Table 3. Standard antibiotics (Ticarcillin, Tetracycline and Gentamicin) showed IZD between 10 ± 0.00 and 30 ± 0.00 mm in all bacterial strains studied. No inhibition zones were found around the disks impregnated with 1% DMSO. These results indicate that zones of inhibition are variable from one bacterial strain to another and from one extract or fraction to another. Considering the ethanolic crude extract of *B. toxisperma* (Bt EtOH Ce), we observed inhibition diameters that ranged from 14 ± 0.47 to 22 ± 0.82 mm. The best diameters were obtained for the *Klebsiella pneumoniae* MDR (IZD 20 ± 0.00 mm) and *Escherichia coli* ESBL (IZD 22 ± 0.82 mm) strains. On the other hand, the lowest inhibition diameters were registered for *Salmonella enterica* (IZD 14 ± 0.47 mm) and *Escherichia coli* ATCC 25992 (IZD 15 ± 0.00 mm). The tests performed on the fractions showed inhibition diameters varying between 9 ± 0.00 mm and 21.33 ± 0.47 mm for the *Escherichia coli* ATCC 25992 and *Klebsiella pneumoniae* MDR strains, respectively. Only the *Escherichia coli* ATCC 25992 strain was sensitive to all fractions and to the crude extract. Fraction F7 showed no inhibition diameter for the *Escherichia coli* ATCC 8739 or *Escherichia coli* ESBL strain. Fractions F2 and F4 showed better inhibition diameters in the majority of strains. This means that the antibacterial compounds of the ethanolic extract of *Baillonella toxisperma* were mainly eluted in the Bt EtOH F2 and Bt EtOH F4 fractions. The minimum inhibitory concentrations (MICs) and minimum bactericidal concentrations (MBCs), reported in Table 4, ranged from 0.31 to 5 mg/mL and 2.5 to 5 mg/mL, respectively. In order to determine the bactericidal or bacteriostatic modes of action of the tested samples, the MBC/MIC ratios were calculated. The results obtained were interpreted as follows: when the MBC/MIC ratio was between 1 and 2, the extract or fraction was bactericidal, and when this ratio was between 4 and 16, the extract was considered bacteriostatic [27]. On this basis, the results in Table 4 show that fraction Bt EtOH F2 is bactericidal against *Escherichia coli* ATCC 8739, *Escherichia coli* ATCC 25992 and *Salmonella enterica* (MBC/MIC ratio = 2) and bacteriostatic against *Klebsiella pneumoniae* MDR and *Escherichia coli* ESBL (MBC/MIC ratio = 4). Fraction Bt EtOH F4 shows bactericidal action in all strains (MBC/MIC ratio = 2). The remarkable antibacterial activity of the ethanolic extract of *B. toxisperma* trunk bark can be attributed to the presence of several phenolic compounds. Indeed, previous studies have demonstrated the antimicrobial and antioxidant activities of some compounds present in the bark of *B. toxisperma*, such as isorhapontin [34,35], biochanin A [36], resveratrol and its derivatives [37,38], epicatechin and epigallocatechin gallate [39,40,41]. An annotation based on mass spectrometry data was also performed on the most active fractions in order to see more clearly the different compounds present and potentially resting antimicrobial activities on the active fractions of *B. toxisperma* (Figure 4).

### 3.4. Analysis of the Most Active Fractions, F2 and F4

The fractions that showed the highest antibacterial activity were analyzed with mass spectrometry. The results of this analysis showed that fractions F2 and F4 had several ion pairs. Fraction F2 showed a majority compound of *m*/*z* 361.092 [C_18_H_16_O_8_^+^] at a retention time (RT) of 2.647 min, which corresponds to rosmarinic acid (1) (Figure 4A,C). Furthermore, the fraction F4 showed three major peaks, *m*/*z* 375.108 [C_19_H_18_O_8_^+^]; *m*/*z* 345.097 [C_18_H_16_O_7_^+^] and *m*/*z* 359.113 [C_19_H_18_O_7_^+^], corresponding to rosmarinic acid methyl ester (2); 3,7,4’,-trimethylquercetin (3) and quercetin 3,3’,4’,7-tetramethyl ether (4), respectively (Figure 4B,D,E,F). The presence of all these phenolic compounds in the F2 and F4 fractions could indeed justify the strong antibacterial activities of the plant *B. toxisperma*. Indeed, previous studies on the plant have shown that polyphenols, tannin and flavonoids are responsible for its antibacterial activities [9]. Several other studies have shown strong antimicrobial activities of rosmarinic acid mainly present in the F2 fraction of *B. toxisperma* [42,43,44,45]. Similarly, several studies have shown antibacterial and antioxidant activities of quercetin and its derivatives [46,47,48]. In view of our results, we can attribute the antimicrobial activity of the F2 and F4 fractions to these different compounds.

### 3.5. Cytotoxicity of the ethanolic crude extract of B. toxisperma

**Figure 5:**
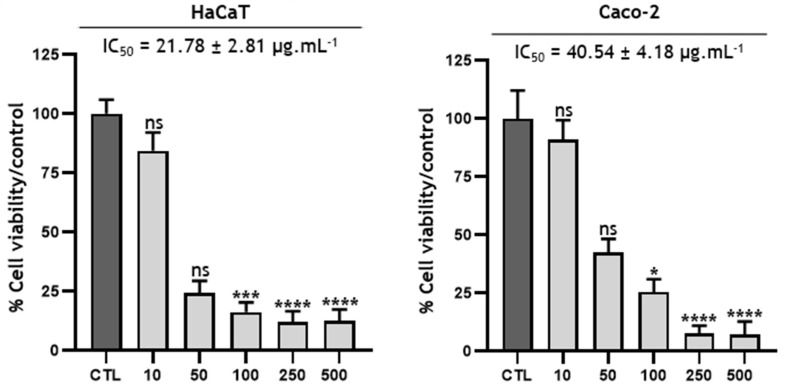
Cytotoxicity of *Baillonella toxisperma* Pierre bark on HaCaT and Caco-2 cells. Statistical tests were performed via reading the Kruskal-Wallis test and Dunn’s post hoc test (ns *p* > 0.9999; * *p* = 0.0304; *** *p* = 0.0007; **** *p* < 0.0001). Differences were observed in comparison with the control (CTL).

The objective of this activity was to evaluate the effect of *Baillonella toxisperma* bark on the overall cell proliferation of two cell lines: HaCaT and Caco-2. The cytotoxic assay was performed at different extract concentrations ranging from 10 to 500 µg/mL (Figure 5). The IC_50_ value was determined from the percentage of cell viability compared to the control. The results show that at up to 50 µg/mL, the *B. toxisperma* extract was well-tolerated by both cell lines. On the other hand, at 100 µg/mL onward, some toxicity was observed in both cell lines. Based on the different IC_50_ values, HaCaT cells seem to be more sensitive than Caco-2 cells, with respective IC_50_ values of 21.78 µg/mL and 40.54 µg/mL. These results seem very consistent not only with the traditional use of *B. toxisperma* but also with the different chemical groups found in the crude extract [49]. Indeed, phenolic compounds are known for their multiple properties: antimicrobial, antioxidant, antiapoptotic, anti-inflammatory and anticarcinogenic [50,51,52,53]. More specifically, certain compounds present in the bark of *B. toxisperma*, such as resveratrol, kelampayoside A, biochanin A and rosmarinic acid, are known to have various beneficial activities on the proliferations of several cell lines [54,55,56,57,58,59]. These compounds may therefore be partly responsible for the observed activity.

## 4. Conclusions

In this study, the antibacterial properties and in vitro cytotoxicity of *Baillonella toxisperma* were investigated using a combined mass spectrometry and molecular array approach. Thus, the combination of these two identification techniques allowed determination, for the first time, of not only the different chemical groups present in this plant but also the molecules potentially responsible for the antibacterial activities of *B. toxisperma*. Among the compounds putatively identified, phenolic compounds such as (i) rosmarinic acid, (ii) quercetin 3,3’,4’,7 tetramethyl ether and (iii) 3,7,4’-tris-*O*-methyl quercetin were identified in the most active fractions. This identification is not exhaustive but brings new information on the chemical composition of *B. toxisperma* with the presence of several polyphenols, such as resveratrol and its derivatives. *B. toxisperma* bark, at very low concentrations, reduces not only the metabolic activity of colon-cancer-cell lines (CaCo-2), but also that of human keratinocyte (HaCaT) cells, which encourages further research on the molecules responsible for this inhibitory activity. All of these results showed therapeutic potential of *B. toxisperma* and justified its use in traditional medicine.

## Figures and Tables

**Table 1:** LC-HRMS determination of compounds in the ethanolic crude extract of *B. toxisperma* in positive ionization mode.

Compound Number	R_T_ (min)	MS (*m*/*z*) [M+H]^+^	Molecular Formula	Fragments	Proposed Identification	Sources	Confidence Level
**1**	1.450	307.081	C_15_H_14_O_7_	307.081, 165.070	Epigallocatechin	PubChem, DNP	3
**2**	1.701	185.084	C_9_H_12_O_4_	185.081, 123.007	Unknown	-	4
**3**	1.710	496.203	C_20_H_30_O_13_	496.203, 295.097, 327.123	Kelampayoside A	PubChem, ChemSpider	3
**4**	1.938	459.092	C_22_H_18_O_11_	459.092, 165.069	Epigallocatechin gallate	PubChem, DNP	3
**5**	2.830	289.071	C_14_H_6_N_7_O	-	Unknown	-	4
**6**	2.851	255.065	C_15_H_10_O_4_	-	Unknown	-	4
**7**	3.550	229.086	C_14_H_12_O_3_	229.086, 165.070	Resveratrol	DNP	3
**8**	3.777	357.133	C_20_H_20_O_6_	357.133, 295.097, 327.123	β-Conidendrin	PubChem	3
**9**	3.427	259.097	C_16_H_18_O_3_	259.097, 152.062, 165.070	3,5-*O*-Dimethyl resveratrol	PubChem, DNP	3
**10**	4.042	243.102	C_15_H_14_O_3_	243.102, 165.069	Methyl resveratrol	PubChem	3
**11**	4.233	420.186	C_21_H_24_O_9_	-	Isorhapontin	PubChem	3
**12**	4.536	316.285	C_18_H_37_NO_3_	-	Unknown	-	4
**13**	5.066	453.337	C_30_H_44_O_3_	453.337, 305.159, 213.165	Unknown	-	4
**14**	5.483	194.117	C_11_H_15_NO_2_	194.117, 163.210	Unknown	-	4
**15**	5.786	238.123	C_16_H_15_NO	-	Unknown	-	4
**16**	6.127	452.322	C_22_H_45_NO_8_	452.322, 294.700, 155.070	Unknown	-	4
**17**	6.278	301.141	C_16_H_18_N_3_O_3_	-	Unknown	-	4
**18**	7.225	487.343	C_31_H_42_N_4_O	-	Unknown	-	4
**19**	7.339	719.306	C_39_H_40_N_7_O_7_	-	Unknown	-	4

**Table 2:** Compounds putatively identified in the molecular network from the ethanolic crude extract of *B. toxisperma*.

MS (*m*/*z*) [M+H]^+^	Molecular Formula	Parent Ion	Cosine Score	Compound Name
307.081	C_15_H_14_O_7_	[M+H]^+^	0.74	Epigallocatechin
291.086	C_15_H_14_O_6_	[M+H]^+^	0.88	Epicatechin
459.092	C_22_H_18_O_11_	[M+H]^+^	0.78	Epigallocatechin gallate
496.203	C_20_H_30_O_13_	[M+NH_4_]^+^	0.83	Kelampayoside A
306.2064	C_15_H_14_O_7_	[M+H]^+^	0.70	Leucocyanidin
322.2379	C_15_H_14_O_8_	[M+H]^+^	0.75	Leucodelphinidin
320.2220	C_15_H_12_O_8_	[M+H]^+^	0.81	Dihydromyricetin
357.133	C_20_H_20_O_6_	[M+H]^+^	0.79	Conidendrin
259.097	C_16_H_18_O_3_	[M+H]^+^	0.76	3,5-*O*-Dimethyl Resveratrol
243.102	C_15_H_14_O_3_	[M+H]^+^	0.72	Methyl Resveratrol
229.086	C_14_H_12_O_3_	[M+H]^+^	0.97	Resveratrol
152.107	C_9_H_13_NO	[M+H]^+^	0.94	2-Amino-1-phenylpropan-1-ol
256.133	C_16_H_17_NO_2_	[M+H]^+^	0.93	N-Benzoyl-L-phenylalaninol
652.115	C_27_H_22_O_18_	[M+NH_4_]^+^	0.96	Corilagin
255.065	C_15_H_10_O_4_	[M+H]^+^	0.81	4’,7-Dihydroxyflavone
438.176	C_21_H_24_O_9_	[M+NH_4_]^+^	0.77	Isorhapontin
285.075	C_16_H_12_O_5_	[M+H]^+^	0.80	Biochanin A

**Table 3:** Inhibition-zone diameters (IZD) of crude extract and ethanolic fractions of *B. toxisperma*.

Inhibition-Zone Diameter (IZD, mm)					
Sample	Bacterial Strains				
	*E. coli* ATCC 25922	*E. coli* ATCC 8739	*K.p* MDR	*S. enterica*	*E. coli* ESBL
Bt EtOH Ce	15 ± 0.00	16 ± 0.47	20 ± 0.00	14 ± 0.47	22 ± 0.82
Bt EtOH F1	12 ± 0.00	13 ± 0.00	10 ± 0.00	Na	9.33 ± 0.47
Bt EtOH F2	13.33 ± 0.94	14.67 ± 0.47	21.33 ± 0.47	16 ± 0.82	19.67 ± 0.47
Bt EtOH F3	11 ± 0.82	11.33 ± 0.94	10.33 ± 0.47	Na	11.33 ± 0.94
Bt EtOH F4	20 ± 0.82	12.33 ± 0.47	14.33 ± 0.47	18 ± 0.47	11.67 ± 0.47
Bt EtOH F5	9 ± 0.00	10.33 ± 0.47	10.67 ± 0.47	11 ± 0.00	10 ± 0.82
Bt EtOH F6	10 ± 0.00	Na	9 ± 0.00	10.67 ± 0.47	Na
Bt EtOH F7	9 ± 0.00	Na	Na	10 ± 0.00	Na
Ticarcillin	19.33 ± 0.47	21.33 ± 0.47	20 ± 0.00	20 ± 0.00	Na
Gentamicin	30 ± 0.00	23 ± 0.00	21 ± 0.00	22.33 ± 0.47	22 ± 0.00
Tetracycline	Na	Na	12 ± 0.00	Na	Na
1% DMSO	-	-	-	-	-

Bt = *Baillonella toxisperma*; Ce = crude extract; *E. coli* = *Escherichia coli*; *K.p* MDR = *Klebsiella pneumoniae* multi-drug-resistant; *S. enterica* = *Salmonella enterica*; *E. coli* ESBL = *Escherichia coli* extended-spectrum beta-lactamase-producing; EtOH = ethanolic; F = fraction; Na = not active.

**Table 4:** Minimum inhibitory concentrations (MICs) and bactericidal concentrations (MBCs) of crude extract and ethanolic fractions of *B. toxisperma*.

MICs and MBCs (mg/mL)										
Sample	*E. coli* ATCC 25922		*E. coli* ATCC 8739		*K.p* MDR		*S. enterica*		*E. coli* ESBL	
	MIC	MBC	MIC	MBC	MIC	MBC	MIC	MBC	MIC	MBC
Bt EtOH Ce	0.62	2.5	1.25	5	1.25	5	1.25	5	1.25	5
Bt EtOH F1	1.25	>5	1.25	5	0.31	2.5	Nt	Nt	2.5	>5
Bt EtOH F2	1.25	2.5	0.62	1.25	1.25	5	1.25	2.5	1.25	5
Bt EtOH F3	2.5	5	1.25	5	1.25	5	>5	>5	2.5	5
Bt EtOH F4	2.5	5	1.25	2.5	1.25	2.5	2.5	5	1.25	2.5
Bt EtOH F5	5	>5	2.5	>5	0.62	5	5	>5	5	>5
Bt EtOH F6	1.25	>5	Nt	Nt	5	>5	2.5	5	Nt	Nt
Bt EtOH F7	5	>5	Nt	Nt	Nt	Nt	2.5	>5	Nt	Nt

Bt = *Baillonella toxisperma*; Ce = crude extract; *E. coli* = *Escherichia coli*; *K.p* MDR = *Klebsiella pneumoniae* multi-drug-resistant; *S. enterica* = *Salmonella enterica*; *E. coli* ESBL = *Escherichia coli* extended-spectrum beta-lactamase-producing; EtOH = ethanolic; F = fraction; Na = not active; Nt = not tested.

## Data Availability

Not applicable.
